# Diagnostic performance of CSF biomarkers in a well-characterized Australian cohort of sporadic Creutzfeldt-Jakob disease

**DOI:** 10.3389/fneur.2023.1072952

**Published:** 2023-02-08

**Authors:** Matteo Senesi, Victoria Lewis, Shiji Varghese, Christiane Stehmann, Amelia McGlade, James D. Doecke, Laura Ellett, Shannon Sarros, Christopher J. Fowler, Colin L. Masters, Qiao-Xin Li, Steven J. Collins

**Affiliations:** ^1^Australian National Creutzfeldt-Jakob Disease Registry (ANCJDR), The Florey Institute of Neuroscience and Mental Health, The University of Melbourne, Parkville, VIC, Australia; ^2^Department of Medicine, Royal Melbourne Hospital (RMH), The University of Melbourne, Parkville, VIC, Australia; ^3^National Dementia Diagnostics Laboratory (NDDL), The Florey Institute of Neuroscience and Mental Health, The University of Melbourne, Parkville, VIC, Australia; ^4^CSIRO Health and Biosecurity, Brisbane, QLD, Australia; ^5^The Florey Institute of Neuroscience and Mental Health, The University of Melbourne, Parkville, VIC, Australia; ^6^The Florey Institute of Neuroscience and Mental Health, Florey Department, The University of Melbourne, Parkville, VIC, Australia

**Keywords:** CJD, tau, Elecsys, RT-QuIC, 14-3-3, CSF

## Abstract

The most frequently utilized biomarkers to support a pre-mortem clinical diagnosis of sporadic Creutzfeldt–Jakob disease (sCJD) include concentrations of the 14-3-3 and total tau (T-tau) proteins, as well as the application of protein amplification techniques, such as the real time quaking-induced conversion (RT-QuIC) assay, in cerebrospinal fluid (CSF). Utilizing CSF from a cohort of neuropathologically confirmed (definite) sCJD (*n* = 50) and non-CJD controls (*n* = 48), we established the optimal cutpoints for the fully automated Roche Elecsys^®^ immunoassay for T-tau and the CircuLexTM 14-3-3 Gamma ELISA and compared these to T-tau protein measured using a commercially available assay (INNOTEST hTAU Ag) and 14-3-3 protein detection by western immunoblot (WB). These CSF specimens were also assessed for presence of misfolded prion protein using the RT-QuIC assay. T-tau showed similar diagnostic performance irrespective of the assay utilized, with ~90% sensitivity and specificity. The 14-3-3 protein detection by western blot (WB) has 87.5% sensitivity and 66.7% specificity. The 14-3-3 ELISA demonstrated 81.3% sensitivity and 84.4% specificity. RT-QuIC was the single best performing assay, with a sensitivity of 92.7% and 100% specificity. Our study indicates that a combination of all three CSF biomarkers increases sensitivity and offers the best chance of case detection pre-mortem. Only a single sCJD case in our cohort was negative across the three biomarkers, emphasizing the value of autopsy brain examination on all suspected CJD cases to ensure maximal case ascertainment.

## Introduction

Creutzfeldt-Jakob disease (CJD) is a rare fatal neurodegenerative disease belonging to a group of diseases known as transmissible spongiform encephalopathies (TSE) or “prion diseases,” which naturally affect humans and other mammalian species. CJD represents the most common prion disease in humans, with ~85–90% of cases occurring sporadically (sCJD) (1). Approximately 10–15% of human prion diseases are inherited, caused by mutations of the prion protein gene (*PRNP*). A minor percentage of human prion disease cases are considered acquired: these include variant CJD (vCJD), a zoonosis linked to bovine spongiform encephalopathy, and recognized to be transmissible through blood transfusion and likely work-place exposure (2), and iatrogenic CJD (iCJD), transmitted through medical interventions. The foundation of all prion diseases is the template directed conversion of normal cellular prion protein (PrP^C^) into abnormally folded conformers (PrP^Sc^), with the continued accumulation and deposition of PrP^Sc^ in the brain (3, 4).

A definite diagnosis of prion disease requires the histological assessment of brain tissue generally and immunodetection of PrP^Sc^ (5). Over the last 20 years, the capacity to diagnose CJD pre-mortem confidently has progressed significantly, due to advances in brain magnetic resonance imaging (MRI) and CSF biomarker detection (5–7). Until recently, 14-3-3 and total Tau (T-tau) proteins in CSF were most commonly applied (8). Both proteins are non-specific markers of neuronal injury or death, which can be measured semi-quantitatively by western blot (14-3-3) or quantitatively by ELISA (both analytes). Based on international experience in carefully selected patients, elevated concentrations of 14-3-3 or T-tau proteins in CSF have ~90% sensitivity and specificity for sCJD (9), although unlike 14-3-3 protein, increased CSF T-tau is not currently part of the World Health Organization (WHO) endorsed internationally recognized diagnostic criteria for CJD (10).

Recently, the development of highly specific diagnostic biomarker assays, based on protein amplification techniques, such as RT-QuIC have improved the confident pre-mortem diagnosis of CJD. The RT-QuIC assay generates through successive rounds of shaking and incubation a detectable signal from miniscule amounts of PrP^Sc^, which act as “seeds” for the template-directed conversion of PrP^C^ into abnormally folded conformers that aggregate (8) and are detected with the fluorescent dye thioflavin T (ThT), which binds to the misfolded multimers (11). Sources of PrP^Sc^ seeds can be from a range of tissues and biofluids, including brain, nasal brushings, skin biopsies and CSF (8, 12–14). RT-QuIC techniques have been modified to shorten assay time and enhance performance (15) with sensitivities up to 95% reported (16), although most experience, especially based on the “first generation” assays report sensitivity of 80–90%, depending on the tissue and substrate utilized (17). However, it is the impressive specificity of the RT-QuIC, approximating 100%, which makes it a vital development in the diagnosis of CJD (8). Overall, the clinical diagnosis of “probable CJD” before death, or in the absence of an autopsy, has been significantly improved through the expansion of the supplementary investigations beyond a typical electroencephalogram (EEG) (18), to include typical brain MRI features (18, 19), CSF 14-3-3 protein detection (5, 6), CSF T-tau protein estimation (20, 21) and most recently the RT-QuIC assay (12, 22, 23).

The present study is a collaboration of the NDDL with the ANCJDR, which aims to determine whether the Roche automated immunoassay Elecsys^®^ platform can improve the precision of T-tau protein CSF estimation, traditionally determined by standardized ELISA (9, 24), and enhance diagnostic sensitivity, including the delineation of an optimized CSF T-tau cutpoint based on pathologically proven sCJD cases and confirmed non-CJD cases. The Elecsys^®^ system has demonstrated good analytical performance and correlation with other available assays, with low variability and reduced testing time (25). In addition, we compared the utility of CSF T-tau vs. 14-3-3 in the diagnosis of sCJD, utilizing the standard CSF 14-3-3 western blot employed in our laboratory, as well as a commercially available (CircuLex™) CSF 14-3-3 ELISA. Finally, we determined the sensitivity and specificity of CSF RT-QuIC in our laboratory utilizing this same cohort of definite sCJD.

## Methods

Study cohorts: Use of CSF in this study was approved by the Human Research Ethics Committee of the University of Melbourne (#1648441). Fifty CSFs from 49 definite sCJD cases (one patient had two separate lumbar punctures) were ascertained through routine ANCJDR surveillance (26, 27); approximately half of the sCJD cohort have been sub-typed, with all three of the sCJD PrP^Sc^ glycotypes according to the Collinge/London nomenclature (28, 29) represented. We used 48 non-CJD cases as controls; 33 cases were originally referred to the ANCJDR as “suspected CJD,” who had an alternative diagnosis achieved through post-mortem examination; 15 PET+ cases consistent with the pathologic changes associated with Alzheimer's disease (AD) were recruited through the Australian Imaging Biomarker and Lifestyle (AIBL) study (30). The clinical classification of the PET+ cases are: 6 probable AD, 3 possible AD, 2 mild cognitive impairment, and 3 cognitive normal. The AIBL PET+ subset is an important disease group, given the frequency of neuropathologically confirmed AD in clinically suspected CJD cases (20). The basic demographics of the sCJD and non-CJD cohorts are shown in Table 1. Forty-eight of the 50 CJD samples were analyzed for 14-3-3 levels; 2 specimens were not tested as they did not meet the suitability criteria (excessive red or white blood cell counts). Whilst the 14-3-3 western blot was carried out on all 48 non-CJD samples, due to insufficient sample volume, the 14-3-3 ELISA was performed on 32 of the non-CJD samples. RT-QuIC was also performed on a subset of the total cohort, which included 41/50 CJD and 44/48 non-CJD cases.

**Table 1:** Basic demographics of the study cohort.

**Diagnosis**	**Total *N* (PM confirmed cases)**	**Age, mean years (range)**	**Sex (M/F)**	**Disease duration, mean months (range)**
Sporadic CJD	50 (50)	68.9 (43–88)	27/23	6.4 (2–24)
Non-CJD	48 (33)	68.2 (38–93)	24/9	NA

PM, post mortem.

Analysis of T-tau protein: T-tau protein concentrations were measured using the automated Elecsys^®^ assay (referred to as Elecsys^®^ Tau) from Roche (Roche Diagnostics Australia, Sydney) according to the manufacturer's instructions as described (25, 31). For comparison, T-tau protein was also measured using a commercially available assay (INNOTEST hTAU Ag, Fujirebio, Ghent, Belgium: referred as INNO Tau) according to the manufacturer's instructions as traditionally employed in our laboratory and described previously (24). The optimal cutpoint for dichotomizing CSF T-tau protein values was determined using receiver operating characteristics (ROC) curve analysis.

Analysis of 14-3-3 protein: CSF 14-3-3 protein was detected through routine diagnostic western immunoblot as described previously (32) with minor technical modifications including the use of pre-cast 12% Bis-tris NuPAGE gels (NP0341, Life Technologies), a monoclonal pan-14-3-3 antibody (SC1657, Santa Cruz), and highly sensitive enhanced chemiluminescence substrate (ECL Prime; GE): each western blot included appropriate pathologically confirmed CJD positive, weak positive, and non-CJD negative controls. The detection of a 14-3-3 immunoreactive band, at or above the pathologically confirmed “weak positive” allowed scoring as positive or negative relative to control CSF. “Atypical positive” results, usually involving the presence of additional banding, are reported to clinicians with a caveat that the result is less typical of sporadic CJD. For the purpose of this study, two atypical results in this cohort (one in the CJD group, and one in the non-CJD group) were classified with the negative results. Two CSF were unsuitable for western blot test and excluded from the receiver operating characteristics (ROC) analysis of the 14-3-3 western and ELISA. CSF 14-3-3 protein concentrations were determined using the commercially available CircuLex™ 14-3-3 Gamma ELISA Kit (MBL Life Science) following the manufacturer's instructions. ROC analysis was performed to obtain an optimal cutpoint on the specificity, without compromising sensitivity. The dataset was also analyzed using the cutpoint defined by Schmitz et al. in their study validating the use of this kit with emphasis on the specificity (33). Additionally, we calculated the sensitivity and specificity using the dichotomising 14-3-3 western blot result using ROC curve analysis.

RT-QuIC assay: CSF samples were analyzed using the RT-QuIC assay as previously described (12, 34) with recombinant full-length Syrian hamster prion protein (Ha rPrP 23-231) as the substrate (34). Based on the ANCJDR and international experience, a CSF sample was considered RT-QuIC positive if at least two out of eight replicate wells had a ThT fluorescence signal higher than the negative control cutoff at the 80 h time point of the assay, with the cutoff value determined as the mean of the relative fluorescence units (rfu) derived from all the negative control sample wells plus four standard deviations (+4SD). CSF specimens were considered negative if none or only one of the eight replicate wells exceeded the determined cutoff value.

Statistical analysis: The AUC value, sensitivity and specificity for Elecsys Tau, INNO Tau, 14-3-3 detection by western blot as well as by ELISA, and RT-QuIC were obtained from Prism 9.

## Results

**Figure 1:**
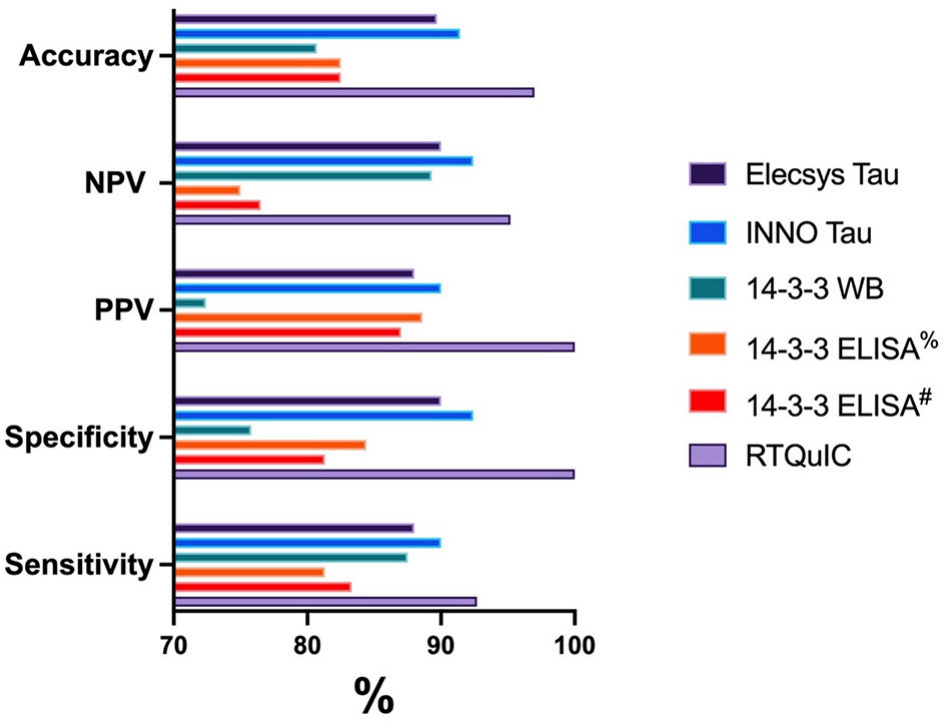
Graphical representation of the diagnostic performance for each biomarker test. ^%^14-3-3 ELISA results based on ROC analysis 23194 AU. ^#^14-3-3 ELISA results based on the previously published cutoff of 20000 AU (33).

Table 2 summarizes the results of CSF testing for Elecsys^®^ Tau, INNO Tau, 14-3-3 western blot, 14-3-3 ELISA and RT-QuIC in sCJD and non-CJD cases. Table 3 and Figure 1 provide a summary of AUC and the diagnostic accuracies for each biomarker test.

**Table 2:** Patient cohorts and results for each CSF biomarker assayed.

		**Tests**					
		**Total** ***N*** **(PM confirmed cases)**	**Elecsys tau (positive/** **negative)**	**INNO tau (positive/** **negative)**	**14-3-3 WB (positive/** **negative/TU)**	**14-3-3 ELISA**^#^ **(positive/** **negative/TU)**	**RT QuIC (positive/** **negative)**
Diagnosis	Sporadic CJD	50 (50)	44/6	45/5	42/6/2	40/8/2	38/3^∧^
	Non-CJD	48 (33)	6/42	5/43	16/32	6/26^∧^	0/44^∧^
	Inflammatory CNS disorder	4 (4)	2/2	2/2	3/1	2/2	0/4
	Leucoencephalopathy	2 (2)	1/1	1/1	1/1	1/1	0/2
	Paraneoplastic/cancer	3 (3)	0/3	0/3	0/3	0/3	0/3
	Drug toxic/metabolic/hypoxia	5 (5)	1/4	1/4	3/2	1/4	0/5
	Neurodegenerative disorders (DLB, cerebellar atrophy, tauopathy, hydrocephalus)	7 (7)	0/7	0/7	4/3	0/7	0/7
	Alzheimer's disease	27^*^ (12)	2/25	0/27	5/22	1/10	0/23

^*^15 of 27 were amyloid β PET+; PM, post mortem; WB, western blot; TU, technically unsuitable CSF sample and thereby excluded from sensitivity and specificity analysis.

^∧^Due to sample shortage, 14-3-3 ELISA was carried out on 32 non-CJD samples, and RT-QuIC was performed on 41 CJD and 44 non-CJD samples.

^#^14-3-3 ELISA positive and negative results based on the previously published cutoff of 20,000 (33).

**Table 3:** Summary of diagnostic utility of CSF biomarkers.

	**Elecsys tau**	**INNO tau**	**14-3-3 WB**	**14-3-3 ELISA^%^**	**14-3-3 ELISA^#^**	**RT-QuIC**
Sporadic CJD (pos/neg)	44/6	45/5	42/6	39/9	40/8	38/3
Non-CJD (pos/neg)	6/42	5/43	16/32	5/27	6/26	0/44
AUC (95% confidence interval)	0.935 (0.88–0.98)	0.921 (0.86–0.98)	0.771 (0.67–0.87)	0.823 (0.72–0.92)	0.881 (0.79–0.97)	0.963 (0.92–1)
Sens/spec	88/87.5	90/89.6	87.5/66.7	81.3/84.4	83.3/81.3	92.7/100
PPV/NPV	88/87.5	90/89.6	72.4/84.2	88.6/75.0	87/76.5	100/93.6
Accuracy	87.8	89.8	77.1	80.7	80.7	96.5

^%^14-3-3 ELISA results based on ROC analysis 23,194 AU.

^#^14-3-3 ELISA results based on the previously published cutoff of 20,000 AU (33).

The CSF Elecsys^®^ Tau has an accurate measurement range of 80–1,300 ng/L. Measured T-tau concentrations for non-CJD samples ranged between < 80 and >1,300 ng/L (4/48 CSFs measured at or below 80 ng/L, and 2/48 CSFs measured above 1,300 ng/L), while sporadic CJD concentrations ranged from 204.8 to >1,300 ng/L (35/50 CSFs measured above 1,300 ng/L). ROC curve analysis defined an optimal cutpoint as 536 ng/L providing 88.0% sensitivity and 87.5% specificity for CJD, and positive predictive values (PPV) and negative predictive values (NPV) of 88.0 and 87.5%, respectively. As a comparison, CSF INNO Tau concentrations (maximum calibrator 2,500 ng/L) for non-CJD samples ranged between 27 and >2,500 ng/L (2/48 CSFs measured above 2,500 ng/L) while sporadic CJD levels ranged from 274 to >2,500 ng/L (34/50 CSFs measured above 2,500 ng/L). ROC curve analysis of the INNO Tau concentrations defined an optimal cutoff as 1,009 ng/L providing 90% sensitivity and 89.6% specificity, with PPV and NPV of 90 and 89.6%, respectively. The diagnostic accuracy was similar between the two assays. The Elecsys^®^ and INNO Tau levels were highly correlated with *r* = 0.95 (Spearman) (data not shown).

14-3-3 protein detection by western immunoblot in this cohort based on dichotomized results, gave 87.5% sensitivity and 66.7% specificity. The PPV and NPV were 72.4% and 84.2%, respectively. As a comparison, using the 14-3-3 ELISA kit to measure 14-3-3 protein in the sub-cohort for non-CJD samples found that the 14-3-3 levels ranged from 1,302 to 221,488 arbitrary units (AU)/ml, with a median value of 6,093 AU/ml; CJD samples ranged from 5,443 t0 161,980 AU/ml, with a median value of 51,251 AU/ml. ROC curve analysis of the 14-3-3 ELISA levels defined an optimal cutoff as 23,194 AU/ml in this cohort, providing 81.3% sensitivity and 84.4% specificity. The PPV and NPV were 88.6 and 75%, respectively. Our results were comparable to the diagnostic utility when applying the published cut point defined by an accredited laboratory (20,000 AU/ml) (33) to our sub-cohort, which gave a sensitivity and specificity of 83.3 and 81.3%, respectively and PPV and NPV of 87 and 76.5%, respectively. As illustrated in Table 3 and Figure 1, the 14-3-3 ELISA slightly improves the diagnostic performance by improving specificity and accuracy compared to the western immunoblot.

The RT-QuIC assay performed at 92.7% sensitivity and 100% specificity. The PPV and NPV were 100 and 93.6%, respectively and the accuracy was 96.5%. All three false negative sCJD samples demonstrated conversion in one well-considered positive (ie 1/8 positive wells was above the cutpoint threshold but the overall result was reported as negative). Interestingly, two of these three cases were sCJD sub-type Type 3 MV, one of which tested positive for 14-3-3 and T-Tau, whilst the other also tested negative for all CJD CSF biomarkers. The third false negative RT-QuIC CSF was also positive for the other CSF biomarkers, and was from a codon 129 valine homozygote, however there was no fresh brain tissue available for sCJD strain typing. Two non-CJD cases, which while ultimately classified negative by RT-QuIC, did also present 1/8 wells with signal above the threshold.

## Discussion

Sporadic CJD incidence in Australia has generally increased over the last 28 years, from an age-standardized mortality rate of ~1.2/million/year in 1993 to ~2/million/year in 2020 (27). This increase is likely due to a combination of factors, such as increased awareness and the access to improved pre-mortem diagnostic biomarkers (27). The ANCJDR receives more than 500 CSF samples per calendar year for investigation of a rapidly progressive neurological or presumed neurodegenerative illness in which sCJD is included in the differential diagnosis (27) and CSF referrals now typically provide just over half of notifications of prion diseases alerted to the ANCJDR annually. Consequently, efficient utilization of the most informative CSF biomarker test battery, including implementation of high-throughput and fully automated testing whenever possible, is helpful to streamline the analysis and reduce the turn-around time of results and focus further investigations.

Detection of 14-3-3 proteins in CSF was never intended as a true screening test for sCJD given the presence of this protein in this biofluid of patients with various alternative acute or subacute serious neurological disorders such as herpes simplex encephalitis and ischaemic injury to the brain (35). Further information can be found on this website (https://www.cjd.ed.ac.uk/sites/default/files/investigations.pdf). Use of the investigation was developed for case confirmation in the setting of a high pre-test likelihood of sCJD: hence, the accuracy of this test, when utilized appropriately, offers good sensitivity and specificity and led to the WHO to include a positive CSF 14-3-3 result in the clinical criteria for sCJD diagnosis in 1998. The ANCJDR has offered CSF 14-3-3 protein detection by western blot since 1997 with good practical utility for surveillance purposes (27). In comparison to western blotting, in our hands ELISA employing the CircuLex™ 14-3-3 Gamma kit offered slightly improved diagnostic accuracy and specificity as previously reported (33, 36) albeit with a minor compromise of sensitivity, with the features of greater quantitation, higher throughput and shorter assay times, offering practical advantages.

Since 2017, analysis of CSF T-tau has been offered by the ANCJDR through the NDDL as part of the diagnostic screen for sCJD (27). Initially, the INNOTEST hTAU Ag ELISA was used with this test requiring considerable manual handling and 2 days for completion. The ELISA T-tau CSF cutoff we determined in this study was similar to that reported in our previous study with a larger cohort (9) and by others (20, 21), supporting that the smaller cohort in the current study was adequate for ROC curve analysis. Since the recent development of the Roche Elecsys^®^ assay for Alzheimer's disease core CSF biomarkers, amyloid beta 1–42, phosphorylated tau at serine 181 (P-tau), as well T-tau (31, 37), T-tau has been measured in the NDDL since 2020 using the Elecsys^®^ Tau assay. The Roche Cobas automated analyzer offers superior precision, an 18 min reaction time, and high throughput capacity over the INNOTEST. Supporting other published results (25), Elecsys^®^ T-tau proved comparable diagnostic accuracy to the previously validated INNOTEST assay (9), as well as marginally superior sensitivity and specificity to 14-3-3 protein detection, confirming its utility for sCJD CSF biomarker screening.

CSF RT-QuIC assay was quickly embraced worldwide as a pre-mortem investigation and has generally become the preferred CSF sCJD biomarker due to its high sensitivity and approaching 100% specificity, especially when compared to non-specific biomarkers such as 14-3-3 or T-tau. The performance of our first generation RT-QuIC assay, with an overall sensitivity of ~93% and specificity of 100% is similar to that reported in studies of larger patient cohorts (17). Although it is accepted that some sCJD molecular subtypes (such as VV2 and MV2K) have lower sensitivity rates (17), these have been recently improved with the use of different recombinant prion protein substrates (15, 38). Additionally, other rarer sporadic human prion disease phenotypes such as variably proteinase sensitive prionopathy (VPSPr) as well as vCJD underperform on CSF RT-QuIC, although alternative protein amplification techniques such as Protein Misfolding Cyclic Amplification (PMCA), in the case of vCJD, can detect prion protein misfolding in tissues (39) and biofluids (40). Importantly, our study has shown that CJD is still a differential diagnosis in a setting of negative RT-QuIC and positive 14-3-3 and/or tau CSF biomarker results.

In this study, RT-QuIC using CSF has high specificity, providing confidence about the accuracy of a pre-mortem diagnosis of sCJD when a result is positive. The very useful, but lower sensitivity of the first generation RT-QuIC complements the non-specific CSF biomarkers (such as T-tau and 14-3-3), as well as autopsy neuropathological assessment; especially in less typical clinical cases or rarer molecular sub-types. Although the overall sensitivity of RT-QuIC was superior to the two non-specific biomarkers in our study, two of the three false negative RT-QuIC results came from samples that did test positive on 14-3-3 and T-tau analysis, highlighting the continued utility of performing all three biomarker assays. Importantly, only one definite sCJD CSF sample was negative on all three CSF biomarker tests. This study supports the proposal to amend the established WHO criteria for the clinical diagnosis of sCJD in a recent review of diagnostic criteria (23).

## Data availability statement

The raw data supporting the conclusions of this article will be made available by the authors, without undue reservation.

## Ethics statement

The studies involving remnant diagnostic CSF from human subjects for research were approved by the Human Research Ethics Committee of the University of Melbourne (#1648441). Written informed consent for participation was not required for this study in accordance with the national legislation and the institutional requirements. The Ethics Committee waived the requirement of written informed consent for participation.

## Author contributions

MS and VL: study design, data collection, data analysis and interpretation, literature search, statistical analysis, creating figures, and writing manuscript. SV: data collection, data interpretation, and manuscript review. CS: data collection, data interpretation, and manuscript editing. AM and LE: data collection and data analysis. JD: statistical analysis and manuscript review. SS and CF: data collection and manuscript review. CM: data interpretation and manuscript review. Q-XL: study design, data analysis and interpretation, literature search, statistical analysis, creating figures, and writing manuscript. SC: review cases included in study and study design, review, and editing of manuscript. All authors contributed to the article and approved the submitted version.
